# Effect of metformin on insulin-resistant endothelial cell function

**DOI:** 10.3892/ol.2015.2883

**Published:** 2015-01-16

**Authors:** HAIYAN CHEN, JIE LI, OU YANG, JIAN KONG, GUANGZHU LIN

**Affiliations:** 1 Cadre Ward, The First Hospital of Jilin University, Changchun, Jilin 130021, P.R. China; 2 Department of Cardiology, The First Hospital of Jilin University, Changchun, Jilin 130021, P.R. China

**Keywords:** metformin, insulin resistance, endothelial cells, nitric oxide, endothelial nitric oxide synthase

## Abstract

The aim of the present study was to investigate the effect of metformin on the function of insulin-resistant (IR) endothelial cells. A model of IR endothelial cells was established by incubating cells with 30 mM glucose, 1 μM dexamethasone and various concentrations of insulin. The nitric oxide (NO) content of the endothelial cells was determined by measuring the rate of nitroreductase production; the endothelin (ET) concentration was examined by enzyme-linked immunosorbent assay; and the expression levels of endothelial nitric oxide synthase (eNOS) were detected using western blotting. The optimal conditions for inducing insulin resistance in endothelial cells were a combination treatment of 10^−4^ mmol/l insulin, 30 mM glucose and 1 μM dexamethasone for 48 h. Notably, metformin administration significantly increased the NO content and reduced the ET-1 concentration in the IR cells compared with the non-treated control cells (P<0.05); furthermore, metformin significantly increased the intracellular eNOS protein expression in IR endothelial cells compared with the non-treated control cells (P<0.05), with an optimal metformin concentration of 10^−3^ mmol/l. Thus, the present study identified that metformin improves the function of IR endothelial cells, possibly through promoting eNOS protein expression and increasing the NO content.

## Introduction

According to global data from the World Health Organization, ~320 million people succumb to diabetes each year and ~70–80% of type 2 diabetic mortalities are due to cardiovascular complications (1). Furthermore, the risk of coronary heart disease is two to three times higher in type 2 diabetes patients compared with normal subjects, with insulin resistance and hyperglycemia identified as important mechanisms in the development of cardiovascular complications in diabetes (2).

Previous epidemiological studies have demonstrated that ~2/3 of acute coronary artery disease or stroke patients exhibit impaired glucose tolerance or diabetes, indicating that abnormal glucose metabolism has a negative impact on endothelial function. Insulin resistance is considered to be the common ground of type 2 diabetes and atherosclerosis (3). In addition, insulin resistance inhibits nitric oxide (NO) synthesis in vascular endothelial cells (4), promotes the secretion of inflammatory mediators, such as tumor necrosis factor, plasminogen activator inhibitor-1 (PAI-1), interleukin-6 (IL-6), C-reactive protein (CRP) and free fatty acid (5,6), and ultimately results in an abnormal blood glucose concentration and dyslipidemia. These pathophysiological conditions consequently lead to atherosclerosis, thrombosis and plaque ruptures, which may cause acute life-threatening cardiovascular and cerebrovascular complications (7).

Metformin is a first-line oral anti-diabetic agent that reduces blood glucose concentration, blood lipid levels, blood pressure and body weight (8). The United Kingdom Prospective Diabetes Study (9) demonstrated that metformin significantly reduced the risk of myocardial infarction by 39% in patients with type 2 diabetes. In addition, it was demonstrated that metformin markedly improved endothelial-dependent vasodilation, whilst reducing the expression levels of dysfunctional biomarkers, such as endothelin (ET)-1, PAI-1, IL-6, and CRP in inflammatory and endothelial cells (10). Furthermore, metformin exhibited a protective effect on blood vessels by ameliorating specific risk factors for cardiovascular disease, and may improve endothelial function by reducing oxidative stress and vascular inflammation, stabilizing atherosclerotic plaques, inhibiting the proliferation of smooth muscle cells and correcting insulin resistance (11). Thus, metformin is widely used in the management of atherosclerosis, in stroke prevention and for inhibiting restenosis following percutaneous transluminal coronary angioplasty.

The formation of atherosclerosis involves endothelial dysfunction, cellular proliferation, the migration of smooth muscle cells, mononuclear phagocytic macrophage differentiation and the formation of foam cells. Among these, vascular endothelial dysfunction is the initiating factor. Current clinical management for atherosclerosis patients includes stents, bypass surgery and stem cell therapy, however, these methods cannot be administrated proactively as vascular stenosis and visceral damage have already caused damage at the time of diagnosis (12). Therefore, correcting insulin resistance and protecting against endothelial dysfunction are current topics of interest (13,14). In the present study, an *in vitro* insulin-resistant (IR) endothelial cell model was successfully established and used to assess the impact of metformin on the protection of endothelial function.

## Materials and methods

### Materials and reagents

The human umbilical vein endothelial cell (HUVEC) line was provided by Dr Ronggui Li of Jilin University (Changchun, China). Trypsin, dimethyl sulfoxide (DMSO; Sigma-Aldrich, St. Louis, MO, USA), fetal bovine serum (Gibco-BRL, Carlsbad, CA, USA) and methyl thiazolyl tetrazolium blue (MTT; GE Healthcare Bio-Sciences, Pittsburgh, PA, USA) were used in the present study. Glucose, NO, and ET-1 assay kits were purchased from Nanjing Jiancheng Biological Products Co., Ltd. (Nanjing, China).

### Establishment of insulin resistance in HUVECs

HUVECs were cultured in DMEM/low glucose (glucose, 5.5 mmol/l) and the third to fourth generations of cultured HUVECs were harvested for use in the present study. To establish the *in vitro* IR endothelial cell model, the cells were divided into nine groups with six replicates per group: Negative control group, the cells were cultured in 200 μl complete medium; insulin-treated groups, the cells were administered with 30 mM glucose, 1 μM dexamethasone and various concentrations of insulin (10^−2^, 10^−3^, 10^−4^, 10^−5^, 10^−6^, 10^−7^, 10^−8^, 10^−9^ mmol/l). The cells were then cultured for 24, 48, and 72 h. Following the defined culture periods, the glucose concentration of the culture media was detected using the glucose oxidase method, according to the manufacturer’s instructions (Nanjing Jiancheng Biological Products Co., Ltd.).

### Effects of metformin on IR HUVEC cells

The present study investigated the effect of metformin on the function of endothelial cells using the IR endothelial cells established as above. The cells were divided into nine groups, each with six replicates: The negative control group, 200 μl normal medium; the model group, IR cells; and, the metformin groups, treated with 10^2^, 10^1^, 10^0^, 10^−1^, 10^−2^, 10^−3^ and 10^−4^ mol/l metformin. After 48 h of culture, 2 μl supernatant was collected from each sample. The glucose concentration was detected using the glucose oxidase method, the NO content was detected using a nitrate reduction assay and the ET-1 concentrations were detected using an enzyme-linked immunosorbent assay kit, according to the manufacturer’s instructions (Nanjing Jiancheng Biological Products Co., Ltd.).

SPSS statistical software (version 17.0; SPSS, Inc., Chicago, IL, USA) was used to process the data by performing an analysis of variance, and a least significant differences test was conducted for pairwise comparisons between the groups. The results were expressed as the mean ± standard deviation. P<0.05 was considered to indicate a statistically significant difference.

### Effect of metformin on the expression level of endothelial nitric oxide synthase (eNOS) in IR HUVECs

Using the optimal concentration of metformin obtained from the above experiments (10^−3^ mmol/l), the present study investigated the effect of metformin on the expression levels of eNOS, using western blotting as previously described (14). BandScan software (Informer Technologies, Inc., Los Angeles, CA, USA) was used to analyze the grayscale, and the eNOS protein expression level was defined as the grayscale ratio of the target protein (eNOS) to the internal reference protein (β-actin). SPSS software (version 17.0; SPSS, Inc.) was used to perform a t-test to compare the expression level of eNOS between the IR + agent-treated group and the IR + agent-free group (negative control group), as well as between the IR + agent-free group and the non-IR group (blank group). P<0.05 was considered to indicate a statistically significant difference.

## Results

### Establishment of the IR endothelial cell model

The IR model was initially established using endothelial cells. Insulin resistance was identified by determining the glucose concentration in the culture media using the glucose oxidase method. Compared with the negative control group, the glucose concentration in the insulin-treated groups (insulin, 10^−4^ mmol/l; glucose, 30 mmol/l; dexamethasone, 1 μmol/l) was significantly increased at 24, 48 and 72 h (P<0.01; Table I). The results of the present study indicate that glucose consumption was reduced and, thus, the IR model was successfully established.

### Protective effect of metformin against IR HUVEC dysfucntion

To investigate the effect of metformin on HUVECs, NO and ET-1 concentration was measured. Compared with the negative control group, a significant difference was identified in the media glucose concentration of cells treated with 10^−3^ and 10^−4^ mmol/l metformin (P<0.01; Table II). This indicates that metformin affects the rate of glucose uptake in HUVECs by increasing their insulin sensitivity. In addition, a significant increase in NO content was identified in the groups treated with 10^−1^–10^−3^ mmol/l metformin (P<0.01; Table II). However, no correlation was identified between the NO and glucose concentration (P>0.05; Table III), indicating that metformin may improve endothelial function directly by increasing the content of NO, independent of changes in the glucose concentration. Furthermore, metformin reduced the ET-1 concentration (P<0.05; Table II). No correlation was identified between the concentration of ET-1 and glucose (P>0.05; Table III), indicating that metformin reduced the endothelial cell damage by decreasing the ET-1 concentration independent of the glucose concentration. The optimal concentration of metformin for improving insulin resistance was 10^−3^ mmol/l. The concentration of NO and ET-1 were negatively correlated (P<0.05; Table III), indicating that NO and ET-1 were mutual restraint factors involved in endothelial cell metabolic processes.

### Effect of metformin on the eNOS protein expression level of IR HUVECs

**Figure 1:**
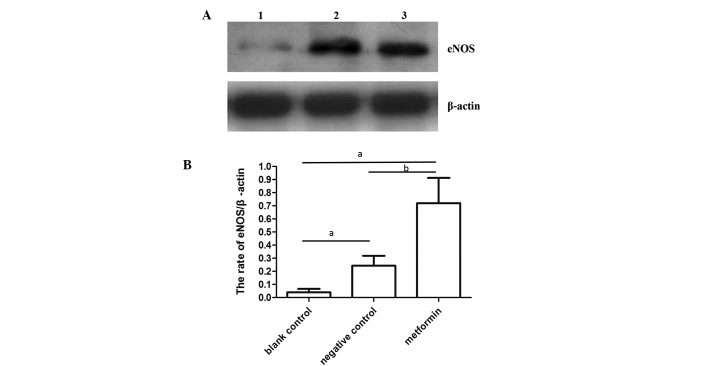
(A) Representative data of the protein expression level of eNOS determined by western blotting. Similar results were obtained from three independent experiments. Lane 1, negative control group (cultured endothelial cells); lane 2, control group (insulin-resistant endothelial cells); lane 3, metformin (10^−3^ mmol/l) group. (B) eNOS protein expression presented as the ratio of eNOS/β-actin. ^a^P<0.01, vs. the blank control group; ^b^P<0.05, vs. the negative control group. eNOS, endothelial nitric oxide synthase.

To investigate the effect of metformin on the expression level of eNOS, western blot analyses were performed (Fig. 1A). Administration of metformin (10^−3^ mmol/l) significantly increased the activity of eNOS when compared with the negative control (IR) and blank control groups (P<0.01; Fig. 1B), indicating that metformin may improve endothelial function by upregulating eNOS expression and, thus, increasing the NO content.

## Discussion

Insulin resistance is associated with endothelial dysfunction and may be a predictor of early atherosclerosis. However, the underlying mechanism by which insulin resistance results in endothelial dysfunction remains controversial. The administration of physiological concentrations of insulin has been demonstrated to stimulate endothelial cells to produce NO, which dilates the blood vessels and increases blood flow in IR patients. However, the reduced insulin sensitivity results in decreased NO production, therefore, weakening the vascular protection provided by NO (15).

The present study established an *in vitro* IR endothelial cell model and investigated the effect of metformin on the protection of endothelial cell function. The results demonstrated that metformin significantly improves glucose uptake in IR endothelial cells, indicating that metformin improves endothelial cell function. Furthermore, to investigate the impact of metformin on endothelial cell function, NO and ET-1 concentration were determined. The optimal concentration at which metformin protects endothelial cell function was 10^−3^ mmol/l. In the metformin-treated cells, the NO level was 2.74±0.42 times higher and the ET-1 concentration was 26.71±1.86% lower, compared with the model (non-treated) group (Table II). In addition, metformin enhanced the activity of eNOS up to 3.11±0.21 times compared with the negative control group and 14.43±2.26 times compared with the blank control group (Fig. 1B).

Metformin is commonly used in clinical practice as a hypoglycemic agent, primarily for the treatment of type 2 diabetes. Various studies have demonstrated that metformin improves the endothelial dysfunction caused by high cholesterol. For example, in one study, long-term use of metformin significantly reduced the risk of stroke and myocardial infarction in patients exhibiting hypercholesterolemia and atherosclerosis (16). Furthermore, Meaney *et al* (17) reported that metformin produced beneficial effects on nitroxidation and endothelial function. Metformin enhanced NO metabolism, reduced CRP and caused various protective endothelial function indices to increase, such as advanced oxidation protein products. Furthermore, O’Hora *et al* (18) demonstrated that metformin directly stimulated NO production by the endothelium.

Endothelial cells regulate vascular tone by releasing vasodilatory factors, such as bradykinin and NO, and vasoconstrictor substances, such as thromboxane A2 and ET (19). Physiologically, the secretion of NO and ET from endothelial cells remains in relatively dynamic equilibrium. However, once endothelial cells are damaged, this equilibrium is disrupted, resulting in various pathophysiological consequences. Metformin may improve endothelial cell function by increasing NO and eNOS levels and reducing the concentration of ET-1, to further protect the endothelial cells against atherosclerosis. However, other studies have demonstrated that metformin improves endothelial cell function by inhibiting the expression of endothelial cell angiotensin II type 1 receptor (17) protecting vascular endothelial function and reducing cardiovascular events in patients with diabetes (20).

In conclusion, the present study demonstrated that metformin enhances endothelial function. Thus, enhancing endothelial function may be one of the mechanisms responsible for the protective effect of metformin in reducing cardiovascular complications in type 2 diabetes. Future *in vitro* studies are required to investigate the specific pathway that is activated by metformin.

## Figures and Tables

**Table I:** Glucose concentration in different endothelial cell groups (n=6; mean ± standard deviation).

Group	24 h	48 h	72 h
Negative control	4.19±0.66	3.78±0.37	3.11±0.64
10^−2^ mmol/l insulin	4.65±0.74^b^	17.44±3.68	29.49±0.54^b^
10^−3^ mmol/l insulin	5.10±0.49^b^	19.74±3.56^b^	28.15±2.28^b^
10^−4^ mmol/l insulin	5.86±0.76^b^	27.58±5.71^b^	33.27±1.63^b^
10^−5^ mmol/l insulin	6.36±2.46^b^	22.55±5.10^b^	29.218±5.76^b^
10^−6^ mmol/l insulin	7.51±1.93^b^	15.48±4.87^b^,^c^	32.23±6.08^b^
10^−7^ mmol/l insulin	6.85±1.71^b^	17.38±4.20^b^,^c^	31.81±4.54^b^
10^−8^ mmol/l insulin	4.77±2.43^b^	15.96±3.30^b^,^d^	15.46±3.90^b^,^d^
10^−9^ mmol/l insulin	5.93±0.58^b^	19.98±2.37^b^,^d^	19.18±5.11^b^,^d^

Cells were treated with 30 mM glucose, 1μM of dexamethasone and various concentrations of insulin.

a P<0.05 and

b P<0.01, vs. the negative control group;

c P<0.05 and

d P<0.01, vs. the 10^−4^ insulin group.

**Table II:** Glucose, NO and ET-1 concentration in endothelial cells (n=6; mean ± standard deviation).

Group	Glucose, mmol/l	NO, μmol/l	ET-1, %
Negative control	4.08±0.51	112.36±1.98	24.56±2.97
Model control	12.97±2.05^b^	39.21±2.21^b^	91.56±1.86^b^
10^3^ mmol/l metformin	5.59±3.11^a^	35.24±3.01	87.31±4.215
10^2^ mmol/l metformin	5.29±1.27^a^	36.84±1.56	90.21±3.86
10^1^ mmol/l metformin	6.46±2.09^a^	38.01±2.99	88.14±2.74
10^0^ mmol/l metformin	6.86±4.33^a^	39.98±1.95	70.63±4.01
10^−1^ mmol/l metformin	5.29±1.42^a^	74.01±4.35^a^	36.43±3.86^a^
10^−2^ mmol/l metformin	5.55±0.85^a^	78.65±3.18^a^	35.27±2.94^a^
10^−3^ mmol/l metformin	5.21±2.02^a^	107.53±2.23^a^	26.71±1.86^a^
10^−4^ mmol/l metformin	5.75±4.15^a^	40.08±4.01	70.16±3.96

a P<0.01, vs. the model control group;

b P<0.01, vs. the negative control group.

NO, nitric oxide; ET-1, endothelin-1.

**Table III:** Correlation between glucose, NO and ET-1 concentration in endothelial cells.

Group	NO/glucose	ET-1/glucose	NO/ET-1
Negative control	No	No	N (r=−0.61)
Model control	No	No	N (r=−0.67)
10^2^ mmol/l metformin	No	No	N (r=−0.69)
10^1^ mmol/l metformin	No	No	N (r=−0.63)
10^0^ mmol/l metformin	No	No	N (r=−0.59)
10^−1^ mmol/l metformin	No	No	N (r=−0.58)
10^−2^ mmol/l metformin	No	No	N (r=−0.51)
10^−3^ mmol/l metformin	No	No	N (r=−0.57)
10^−4^ mmol/l metformin	No	No	N (r=−0.56)
10^−5^ mmol/l metformin	No	No	N (r=−0.52)

N, negative correlation; P, positive correlation (P<0.05). NO, nitric oxide; ET-1, endothelin-1.
